# A systematic scoping review of change management practices used for telemedicine service implementations

**DOI:** 10.1186/s12913-020-05657-w

**Published:** 2020-09-01

**Authors:** Joanna Kho, Nicole Gillespie, Melinda Martin-Khan

**Affiliations:** 1 UQ Business School, The University of Queensland, Colin Clark Building 39 Blair Drive, St Lucia, Brisbane, QLD 4072 Australia; 2 Centre for Health Services Research, The University of Queensland, Brisbane, Australia

**Keywords:** Telemedicine, Telehealth, Virtual care, Change management, Organizational change, Change readiness, Resistance, Implementation

## Abstract

**Background:**

Telemedicine improves access to health care services enabling remote care diagnosis and treatment of patients at a distance. However, the implementation of telemedicine services often pose challenges stemming from the lack of attention to change management (CM). Health care practitioners and researchers agree that successful telemedicine services require significant organizational and practice change. Despite recognizing the importance of the “people-side” of implementation, research on what constitutes best practice CM strategies for telemedicine implementations remains fragmented, offering little cohesive insight into the specific practices involved in the change process. We conducted a systematic scoping review of the literature to examine what and how CM practices have been applied to telemedicine service implementation, spanning a variety of health care areas and countries.

**Methods:**

Three bibliographic databases (CINAHL, PubMed, and ISI Web of Science) and four specialist telehealth journals were searched. To keep the review manageable and relevant to contemporary telemedicine technologies and contexts, the search was limited to articles published from 2008 to 2019. Forty-eight articles were selected for inclusion.

**Results:**

From the 48 articles, 16 CM practices were identified relating to either strategic or operational aspects of telemedicine implementations. We identify the key CM practices that are recognized in the broader CM literature as essential for successful and sustained change but are not commonly reported in telemedicine implementation studies. We draw on the CM literature to provide a comprehensive process-based, researched-informed, organizing framework to guide future telemedicine service implementations and research.

**Conclusions:**

Our findings suggest that the slow rate of adoption of telemedicine may be due to a piecemeal approach to the change process, and a lack of understanding of how to plan, manage and reinforce change when implementing telemedicine services.

## Background

Over the past few decades, health care organizations have been undergoing significant organizational and practice change to incorporate information communication technologies (ICTs), with the aim of reducing costs, improving quality, increasing efficiency and effectiveness and raising patient or client satisfaction [1, 2]. The incorporation of telemedicine services is a prominent example of such change. Telemedicine is the delivery of health care services using ICTs as a substitute for traditional face-to-face interactions between patient and provider, enabling the remote care, diagnosis and treatment of patients at a distance [3]. Telemedicine is increasingly valued for providing health care services to patients, either for those with limited access to specialist assessment or management of care needs due to remoteness, convenience or managing patient flow [4].

The use of telemedicine has increased significantly over time [5], however, research on telemedicine uptake indicates it still remains low as a percentage of all care, with high rates of non-participation by different stakeholders [6, 7]. While limited reimbursement and current licensure laws pose barriers to the widespread use of telemedicine [8, 9] slow adoption has also been attributed to human factors [10, 11], organizational issues [12, 13] and cultural barriers [14]. The success of telemedicine rests not only on resolving technical, regulatory and financial issues but also on the management of human and organizational change [15]. Successful long- term implementation requires sustained resolution of all these factors concurrently.

Change management (CM) is often recognized as integral to the implementation of telemedicine [16, 17], yet is often approached in an ad-hoc, sporadic and reactive way, and reported as “lessons learned” when retrospectively evaluating a service implementation [15, 18, 67]. CM takes a systematic approach based on “an enabling framework for managing the people side of change” [19] that involves a set of processes, practices and deliberate activities intended to facilitate and guide an organization to move from its present state to a desired future state [20].

Researchers studying telemedicine implementation typically frame their understanding of the challenges and factors influencing uptake by identifying and listing “barriers” and “enablers” [21, 22]. For example, some enablers cited are the development of organizational protocols, adequate funding and support, user training plans and change management plans [16]. Common barriers reported are lack of technological compatibility, resistance to change, lack of adequate reimbursement, lack of usability and medico-legal and liability concerns [8, 16]. The assumption underlying this work is that telemedicine adoption can be achieved by increasing the enablers and reducing the barriers [22]. Yet, many barriers to telemedicine remain stable over time, with studies continuing to report the same barriers with little signs of improvement [23].

Some authors have focused on tools to assess telemedicine ‘readiness’ [24] or constructed models to explain clinician ‘acceptance’, recognising that clinician acceptance has the greatest influence on uptake and sustainability [25]. While these current telemedicine change acceptance and readiness tools and models are helpful, a limitation is that they do not account for or guide the process of change involved in implementing and adopting telemedicine services. Furthermore, because most telemedicine studies reported in the literature involve trials, feasibility studies or pilots [17, 23, 26], the focus is on short-term adoption and project management, or the technical side of implementing telemedicine services (i.e., technical aspects that show how to design, develop and deliver a service) [23].

As telemedicine is a “key solution to resolve both contemporary and future challenges in health care and social care” [17], there exists a number of guidelines, implementation resources and tool kits aimed to facilitate the implementation of telemedicine solutions [17, 27]. For example, a ‘MOMENTUM’ report put together by various European interest groups and stakeholders, identifies 18 critical successful factors to guide the deployment of telemedicine in routine delivery of health services on a large scale [17]. Similar to most guidelines and recommendations, ‘preparing and implementing a CM plan’ is considered a critical success factor. However, due to limited understanding of what CM is and how to apply it, CM plans are often poorly executed or not implemented at all.

While the importance of CM for successful telemedicine implementation is known [13, 16, 17, 28], the literature on CM application in telemedicine remains fragmented, lacking consolidated detail about how they are applied, the frequency of practices used, and the outcomes produced when guiding and implementing change. In other words, the literature on telemedicine services lacks an integrated framework that provides cohesive learning and insight about CM practices reported in telemedicine implementation studies. Such a review and synthesis of extant literature is important for informing and advancing theory, research and practice on the effective implementation of telemedicine services.

Therefore, this study aims to: (1) identify what CM practices have been applied to the implementation of telemedicine services; (2) identify the frequency of the CM practices reported; (3) provide a CM practice framework specific for telemedicine implementations and; (4) identify the gaps in the current CM approach to implementing telemedicine, as reported by existing literature, by comparing it to the broader CM literature.

## Methods

To assess the CM practices used for implementing telemedicine services we conducted a scoping study of the avaliable research literature. While keeping our search broad, we aimed to conduct a comprehensive, reproducible, and systematic search of published literature. Scoping reviews involve five steps or stages: (1) identifying the research question/s; (2) identifying relevant studies; (3) study selection; (4) charting the data; and (5) collating, summarizing and reporting the results [29]. We followed Arksey and O’Malley’s [29] approach guided by Tricco and colleagues’ PRISMA-ScR checklist (Preferred Reporting Items for Systematic reviews and Meta-Analyses extension for Scoping Reviews) [30]. As scoping reviews are not eligible for registration or inclusion in databases of systematic review protocols [31], we first reviewed existing telemedicine literature to find that no other published scoping or systematic review focused on our topic of interest. Performing a scoping study was a useful way of mapping key concepts that underpinned our research topic that had not been comprehensively reviewed [32, 33].

### Identifying relevant studies

We conducted a search of the peer-reviewed literature using three electronic bibliographic databases: CINAHL, PubMed, and ISI Web of Science. In addition, we searched the four most cited telehealth journals: *Journal of Telemedicine and Telecare, Telemedicine Journal and e-Health, Telemedicine and e-Health,* and *Telemedicine Journal* (the last three journals all sourced via Mary Ann Liebert Publishers Journals database)*.* Reference lists of included articles were also systematically searched, for additional relevant studies. The search strategy, terms and database selection were reviewed and determined in consultation with a university librarian (with expertise in literature reviews and searches), and the third author (an experienced health scientist and health administrator). When applicable, expanders were used to broaden the scope of our search. A broad range of search terms associated with our topic were used, guided in part by the MeSH heading terms (and associated entry terms) of Telemedicine, Change Management and Pilot Projects. Table 1 outlines the search strategy and terms used for each database and journal. During pilot testing of the search strategy, some terms or phrases (such as “attitude” and “attitude to change”) were removed from the final search because the term was either not useful (i.e., did not generate any additional studies) or could not be found in some databases.

**Table 1:** Search strategy by database and journals

Database/journal and field selection used	Search strategy and terms
CINAHL (via EBSCOhost) Field selection: “TX All Text”	(telemedicine OR telehealth OR telecare OR mhealth OR m-health OR ehealth OR e-health) AND (pilot OR adopt* OR implement*) AND (“change management” “organi?ational change” OR readiness OR resist*)
PubMed Field selection: “MeSH Terms” (for terms telemedicine, “pilot projects” and “change management”) Field selection: “Text Word” (for all other terms)	Search #1: (telemedicine OR telecare OR m-health OR e-health) Search #2: (“pilot projects” OR adopt* OR implement*) Search #3: (“change management” OR “organizational change” OR readiness OR resist*) Combine search: #1 AND #2 AND #3
Web of Science (core collection) Field Tag: “TS = Topic”	Set #1: TS = (telemedicine OR telehealth or telecare OR mhealth OR m-health or ehealth or e-health) Set #2: TS = (pilot OR adopt* OR implement*) Set #3: TS = (“change management” OR “organi?ational change” OR readiness OR resist*) Combine sets: #3 AND #2 AND #1
Mary Ann Liebert Publishers Journals (Journals: Telemedicine Journal and e-Health, Telemedicine and e-health and Telemedicine Journal) Field selection: “Anywhere”	(telemedicine OR telehealth OR telecare OR mhealth OR m-health OR ehealth OR e-health) AND (pilot OR adopt* OR implement*) AND (“change management” OR “organi?ational change” OR readiness OR resist*)
Journal of Telemedicine and Telecare (JTT) (via SAGE journals) Field selection: “Anywhere”	(telemedicine OR telehealth OR telecare OR mhealth OR m-health OR ehealth OR e-health) AND (adopt* OR implement* OR pilot) AND (“change management” OR “organi?ational change” OR readiness OR resist*)

To keep the review manageable and reflective of current issues concerning contemporary technology, telemedicine services and organizational contexts, the search was limited to articles published between January 2008 and June 2019. We also applied limiters in order to select papers written in the English language and that were peer-reviewed. Articles obtained through our search were imported and stored in Endnote (a reference management software used to manage references) for screening.

### Study selection

Peer-reviewed, empirical articles were reviewed if they met the following criteria: (1) examined health care services using ICTs (e.g., videoconference or store-and-forward systems) that enable virtual interactions between patient and provider (e.g., medical and allied health practitioners) for remote care, diagnosis and/or treatment of patients at a distance; (2) reported or described the evaluation of pilot studies and/or implemented telemedicine services and; (3) referred to the use of CM strategies during the implementation and adoption of services. Literature reviews, systematic reviews, conceptual papers and discussion pieces were excluded; however, their reference lists were reviewed for relevant empirical studies. Similarly, reference lists of articles mentioning some form of telemedicine service were also reviewed for additional sources. Conference and poster abstracts and news articles were excluded during the screening process. Team discussions with all authors were held throughout the review process to discuss decisions regarding the study inclusion and exclusion list, which was refined accordingly based on the abstracts and full articles retrieved from the search.

During the identification process, the first author JK independently reviewed titles, abstracts and full articles by categorizing each article into an ‘included’ or ‘excluded’ group. Papers that clearly met the inclusion criteria based on title and abstract review were subjected to a full text review and on this basis sorted into the included or excluded group. Articles that did not clearly meet the inclusion criteria or where the reviewer was uncertain about its eligibility were set aside for team discussions with all authors. Specifically, questions and challenges regarding article eligibility and uncertainty in the application of the inclusion criteria were resolved through further review and discussion by the authors until consensus was reached. Several team meetings were held throughout the review process to resolve ambiguity related to study selection and to ensure that full articles were relevant for inclusion.

### Data charting process

Following the framework of Arksey and O’Malley [29], we extracted data to inform our research aims using NVivo 12, a qualitative data analysis software designed to help organize, store and analyse data. Included articles were imported into NVivo. General information about each article was then charted [29] and was categorized into specific ‘codes’ or categories. Data included the author(s), year of publication, type of telemedicine service, modality (e.g., video conference) and country of implementation, as well the outcome of the implementation (e.g., successful or not successful). The frequency of CM practices reported for each study was also recorded.

We then analyzed the included articles that reported specific CM practices used when implementing telemedicine services. We extracted CM activities relating to the facilitation of telemedicine implementation, which corresponded with the 10 change steps commonly associated with prescriptive change models [20]. Each identified CM activity was then clustered and coded into broader categories, resulting in a total of 16 specific change practices. Guided by the causal model of organizational change developed by Burke and Litwin (1992), we noted that CM practices identified in our analysis related to either transformational factors of change involved with strategy and leadership or; transactional factors concerned with the day-to-day operations of a change [34]. As such, we further categorized these practices as: 1) *strategic practices*: practices used to direct and promote change and build alliances when implementing telemedicine services; or 2) *operational practices*: practices used to manage the impact of the change on the day-to-day operations of telemedicine services.

In line with the recommendations from Arksey and O’Malley [29], we did not assess the methodological quality of the included studies. Scoping reviews are less restrictive than systematic reviews enabling a broader range of study designs to be included, rather than limiting inclusion based on research quality [29, 33]. Importantly, given the heterogeneity of the existing evidence base of the field, and the fact that the large majority of studies are written as programme descriptions and case reports, a formal quality criteria could not be applied [35].

### Synthesis of results

Guided by Arksey and O’Malley’s approach [29], an analytical framework was used to collate and present our findings. First, we created a data table (i.e., Table 2) for our study characteristics (i.e., author(s), year of publication etc.).

**Table 2:** Characteristics of selected studies

First author (Year)	Type of telemedicine service	Modality	Country of implementation	# of CM practices	Outcome
1. Adler et al., 2014 [18]	Telemental health → VA rural community-based outpatient clinics	VC	South Central, USA	3	S
2. Alkmim et al., 2015 [36]	Primary care → municipalities	VC	Brazil, South America	6	S
3. Avey et al., 2013 [37]	Telepsychiatric services → rural clinics	VC	Alaska, USA	12	S
4. Bagot et al., 2017 [38]	Neurological services → regional hospitals	VC	Victoria, Australia	9	S
5. Bagot et al., 2020 (first online in 2018) [39]	Neurological services → regional hospitals	VC	Victoria, Australia	2	S
6. Bhaskaranarayana et al., 2009 [40]	Range of specialist services → rural areas	VC, S&F	India	5	S
7. Blanchet, 2008 [41]	Range of specialist and educational services → rural areas	VC	Washington, USA	4	S
8. Brooks et al., 2012 [42]	Telemental health → rural American Indian Veterans	VC	Western USA	11	S
9. Cadilhac et al., 2014 [43]	Neurological services → delivered to regional hospitals	VC	Victoria, Australia	8	S
10. Cain et al., 2016 [44]	Surgical specialist care → surgical patients in army clinics	VC	Landstuhl, Germany	5	S
11. Chipps et al., 2012 [45]	Telepsychiatry consultation → regional hospitals	VC	KwaZulu-Natal, Africa	10	S
12. Cifuentes et al., 2016 [46]	Telepediatric services → primary care hospitals	VC	Bogota, South America	1	NR
13. Davis et al. 2017 [47]	Tele-Intensivist →Military community	RM, VC	USA	1	S
14. Doolittle et al., 2019 [48]	TeleHospice (palliative care) → rural communities	VC	Kansas, USA	6	S
15. Doorenbos et al., 2011 [49]	Medical education, case conferences and telepsychiatry consultations → rural communities	VC	Washington and Alaska, USA	10	S
16. Ganapathy et al., 2016 [50]	Range of specialist services → remote hospitals	VC, S&F	Kaza/Keylong, India	7	S
17. Ganapathy et al., 2019 [51]	Tele-emergency services → remote hospitals	VC, S&F	Kaza/Keylong, Northern India	3	S
18. Ganapathy et al., 2020 (online in 2019) [52]	Teleconsultations, screening services for noncommunicable diseases → regional areas	VC, S&F	Six regions in India	5	S
19. Hines et al., 2015 [53]	Tele-speech pathology → rural schools	VC	Sydney, Australia	4	S
20. Janardhanan et al., 2008 [54]	Teledermatology services → nursing homes	S&F	Singapore	4	S
21. Jury et al., 2013 [55]	Telepaediatric services → patients at home	VC	Melbourne, Australia	13	S
22. Kassam et al., 2012 [56]	Teleopthalmology services → remote clinics and in-house	S&F	Alberta, Canada	4	S
23. Kim et al., 2013 [57]	Telepsychiatry services → primary care and other health care organizations	VC	Gulf Coast/Atlanta, USA	7	S
24. Latifi et al., 2014 [58]	Range of specialist services → inhabited islands	VC, S&F	Cabo Verde, Sub-Saharan Africa	7	S
25. Latifi et al., 2016 [59]	Range of specialist services → regional hospitals	VC	Albania, Europe	9	S
26. Lindsay et al., 2015 [60]	Telemental health services → rural Veteran Affairs clinics	VC	South Central USA	8	S
27. Lowery et al., 2014 [61]	Range of specialist services → rural community hospitals	VC	Arkansas, USA	4	S
28. Martinez et al., 2017 [62]	Range of health care providers → Veteran Affairs facilities	VC	USA	10	NR
29. Odor et al., 2011 [63]	Telepsychiatry services → clinics of underserved communities	VC, S&F	California, USA	5	S
30. Pare et al., 2016 [64]	Telepathology services → remote hospitals without pathologists on-site	S&F	Quebec, Canada	9	S
31. Quanbeck et al., 2018 [65]	Primary care and other health care providers→ rural patients	RM	Wisconsin, USA	12	NS
32. Rufo, 2011 [66]	Tele-intensivists → acute care facilities, outreach sites	RM, VC	Illinois, USA	9	S
33. Sanabria et al., 2012 [67]	Range of specialist services → rural health care facilities	VC, S&F	Valenzuela, South America	7	S
34. Saurman et al., 2014 [68]	Telemental health emergency services → remote and regional areas	VC, T	New South Wales, Australia	4	S
35. Schettini et al., 2019 (first online in 2017) [69]	Nephrology e-Consult program → primary care providers	S&F	North Carolina, USA	3	NR
36. Scott et al., 2012 [70]	Specialist care advice to treat complex chronic health conditions → rural primary care providers	VC	Pacific Northwest, USA	4	S
37. Sharma et al., 2011 [71]	Telerehabilitation speech pathology → patients	VC	Queensland, Australia	1	S
38. Shaw et al., 2013 [72]	Primary care → Veteran Affairs clinics	T	USA	7	S
39. Shiferaw et al., 2012 [73]	Teledermatology, teleradiology and telepathology services → remote areas	S&F	Ethiopia, Africa	6	NS
40. Singh et al., 2010 [74]	Range of health care services → rural areas	VC	Georgia, USA	5	S
41. Stevenson et al., 2018 [75]	Specialist care advice to treat common chronic illnesses → remote primary care providers	VC	USA	10	S
42. Stronge et al., 2008 [76]	Teledermatology → army clinics	S&F	USA	4	NR
43. Taylor et al., 2015 [77]	Palliative care, home-based rehabilitation and geriatric services → the home	VC, RM	Adelaide, Australia	6	NR
44. Taylor et al., 2016 [78]	Primary health care → community health services	RM	England	10	S
45. Tetu et al., 2012 [79]	Telepathology diagnostic services → regional or University hospitals	VC	Eastern, Quebec (Canada)	2	NR
46. Visser et al., 2009 [80]	Telepaediatric physiotherapy services → regional communities	S&F (video clips)	Netherlands	5	NR
47. Waugh et al., 2018 [81]	Telemental health services → urban primary care clinic (VC)	VC	Colorado, USA	11	S
48. Wood, 2011 [82]	Tele-ICU → community hospitals	VC, RM	Massachusetts, USA	3	S

*Abbreviations*: *VC* Videoconferencing systems, *S* Successful, *S&F* Store and forward systems, *NS* Not successful, *RM* Remote monitoring, *NR* Not reported, *T* Telephone

**Fig. 1:**
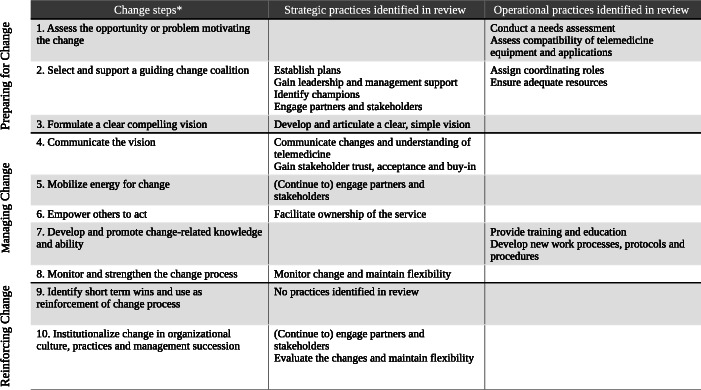
Change management process and practices reported in telemedicine service implementation studies. *Adapted from [20]

Second, we developed a figure that captured a consolidated overview of the CM practices that were identified in our review and matched each one to a corresponding change step commonly associated with established prescriptive change models. We then organized the CM practices further to show whether they were associated with either strategic or operational practices. A process approach for organizational change [20] was then depicted, by organizing the change steps and strategic and operational CM practices using the Prosci 3-Phase Change Process – *preparing for change*, *managing change* and *reinforcing change* [19]. Figure 1 helped us to identify which CM practice/s had been commonly neglected, thus identifying gaps in the current approach to implementing telemedicine, as reported in the existing literature (i.e., addressing research aims three and four).

Third, we produced another table that provided examples associated with each CM practice identified in our review to show how a specific practice was used. We also included the studies which referenced each CM practice to show the frequency of the CM practice reported in literature. Table 3 thus addressed aims one and two.

**Table 3:** Summary table of change management practices reported in telemedicine literature with examples and related articles that refer to its application

Change Management Practice	Examples of CM Practice	Articles that mentioned applying CM Practice
1. Conduct a needs assessment	Assess organizational characteristics, readiness and needs of the clinics and providers to inform design process	[3, 10, 11, 14–16, 20, 23, 25, 29–31, 59, 60, 65]
2. Assess compatibility of telemedicine equipment and applications	Consider other services being provided, existing infrastructure, new technology and appropriate location for telemedicine equipment	[8, 11, 20, 27, 33, 55, 59, 65, 75, 76]
3. Establish plans	Collaborate with key stakeholders to plan and design telemedicine services	[2, 3, 6, 8, 11, 15, 23, 25, 29–31, 59, 65]
4. Gain leadership and management support and commitment	Provide direction through influence to bring about change	[4, 14, 20, 22, 24, 25, 27, 31–33, 58–60, 64, 74]
5. Identify champions	Select key staff to promote, legitimize and build awareness about telemedicine services	[3, 4, 11, 20, 22–24, 26, 27, 30, 33, 60, 64, 74, 75]
6a. Engage partners and stakeholders	Involve stakeholders in design process through frequent communication and building relationships and alliances	[3, 4, 6, 9, 10, 14–16, 20, 23, 24, 27, 29–33, 35, 60, 64, 65, 73, 75, 76]
6b/6c. (Continue to) engage partners and stakeholders	Continue engaging stakeholders to reaffirm value of telemedicine and to obtain feedback	[4, 9, 15, 30, 32, 60, 65]
7. Develop and articulate a clear, simple vision	Have a shared vision with partners and stakeholders	[4, 20, 22, 32, 62, 64]
8. Assign coordinating roles	Assign telemedicine coordinators at both participating and provider site	[1, 3, 4, 7, 9–11, 15–18, 21, 22, 24, 25, 27, 31, 37, 67, 75]
9. Ensure adequate resources	Complete a workflow analysis to ensure adequate resources are deployed to support telemedicine services	[1, 3, 4, 8, 11, 17, 20, 22, 26, 27, 58, 65, 67, 75, 82]
10. Communicate changes and understanding of telemedicine	Disseminate information about changes, benefits, limitations of telemedicine and raise awareness	[2–4, 6–9, 11, 16, 18–20, 24, 25, 27, 31, 32, 34, 58, 60, 64, 73, 75]
11. Gain stakeholder trust, acceptance and buy-in	Build confidence and familiarity for the new system and conduct regular site visits to provide education	[3, 7, 8, 10, 11, 15, 19, 20, 24–27, 30–32, 34, 35, 55, 59, 60, 62, 65, 74, 75]
12. Facilitate ownership of the service	Allow users as choose how and when service should be utilized to facilitate ownership	[9, 14, 20, 23, 24, 32, 33, 73]
13. Provide training and education	Training includes how to use equipment, troubleshoot and how to conduct consultations through the technology	[1–3, 6–9, 11, 12, 14–16, 18, 19, 21–25, 27, 31–34, 55–60, 67, 73, 75, 76, 82]
14. Develop new work processes, protocols and procedures	Develop guidelines and clinical protocols. Customize existing workflow to accommodate the use of telemedicine services	[2–4, 6, 8–11, 13, 15, 18–24, 26, 27, 29–31, 34, 35, 59, 60, 62, 73, 75, 76, 82]
15. Monitor change and maintain flexibility	Refine services by obtaining periodic feedback through reporting systems and regular meetings with stakeholders	[2–5, 8, 15–18, 25, 29–32, 55, 59, 60, 64, 67, 73, 75–82]
16. Evaluate the changes and maintain flexibility	Evaluate patient outcomes, quantify efficiency, assess the capacity of telemedicine operations and conduct a cost analysis	[2, 4, 5, 8, 11, 15, 16, 20, 22, 31, 59, 60, 76, 82]

Article numbers presented in this table aligns with the studies identified in this review listed in Table 2

Collectively, this approach provided a consolidated overview of what is known about CM practices for implementing telemedicine services.

## Results

### Main characteristics of the selected articles in review

**Fig. 2:**
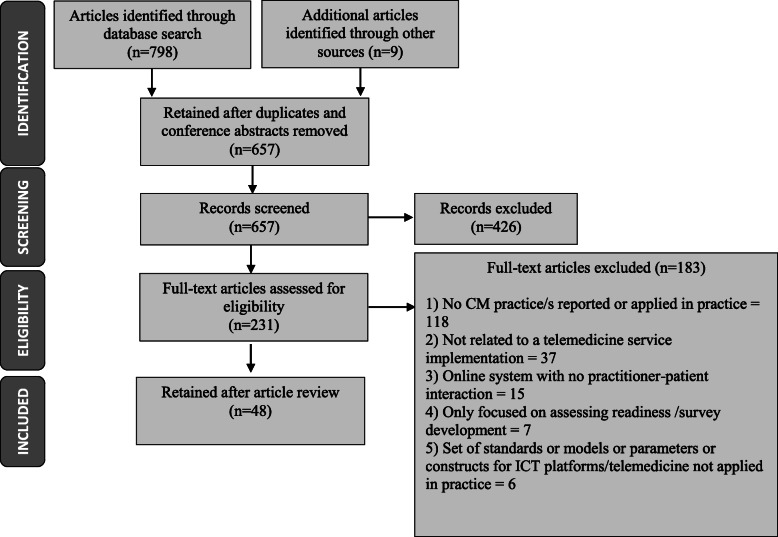
Schema portraying results of the literature search and selection for inclusion

The database search resulted in 798 articles. Additional articles (*n* = 9) identified through other sources (e.g., reference lists) were added. As shown in Fig. 2, after removal of duplicates, the number of articles reduced to 657 articles. If the abstract met the eligibility criteria or if the relevance of the study was unclear from the abstract, then a full-text review was completed. A total of 231 full-text articles were reviewed and 183 of these articles were excluded, retaining 48 articles for analysis. The selected studies were then stored, coded and managed using NVivo 12. As explained in detail in Fig. 2, common reasons for study exclusion included: (1) articles focused on social or economic changes, or individual-level or patient changes in health behaviors, and patient or clinical outcomes; (2) articles that explored change recipients’ perspectives and experiences regarding the barriers or challenges before and during the implementation process with no mention of how these barriers were addressed by using change management practices; (3) guidelines, recommendations or specific strategies that were mentioned in hindsight or as ‘lessons learned’, thus not a ‘tried and tested’ CM practice or applied in practice during an implementation of a telemedicine service, which was the focus of our review.

The majority of telemedicine service implementations reported in the literature were from the United States of America (46%), followed by Australia (17%), Asia (10%), Europe (8%) and the rest from South America, Canada and Africa (6% each). Most studies were based on real-time telemedicine, such as videoconferencing (VC) systems (54%). Others delivered services through a mix of VC and store-and-forward (S&F) systems (17%), VC and remote monitoring (RM) (8%) and VC and phone (1 study). While the rest used RM only (4%), S&F delivery only (13%) and phone only (1 study). The selected articles identified in this review reported on a range of specialist services, with 12 studies focusing specifically on mental health services (e.g., psychiatric and neurological services), while other articles focused on primary care, palliative care, surgical care, intensive care, nephrology, pathology, pediatrics, dermatology and geriatric care services. Table 2 shows a summary of study characteristics.

The most CM practices mentioned in a single study was 13/16 [55]; two studies included 12/16 CM practices [37, 65]; seven studies reported between 10 and 11 practices, 15 studies mentioned six to nine practices; while the remaining studies (*N* = 23) reported one to five practices used to implement change (see Table 2).

Of the 48 studies included in this review, 32 (84%) articles reported successful outcomes, suggesting that telemedicine services were successful and ongoing during the time of the study and/or services had since expanded to other regions or sites. Five studies (13%) did not report on success or whether trials progressed past the pilot stage or evolved to an ongoing service. Only two studies in this review reported a non-successful outcome due to poor uptake of services and a depletion of funding, resulting in declined use by both clinicians and patients [65, 73] (see Table 2).

### Change management practices identified in review

#### Phase 1. Preparing for change – strategic practices

Our review identified five strategic practices that are important during the preparatory phase of the change process. These practices centred on selecting and supporting a guiding change coalition and formulating a clear compelling vision.

##### Establish plans

Fourteen of 48 studies reported on the importance of planning for the establishment of telemedicine services. Strategic planning involved the collaboration of a number of key stakeholders (i.e., clinicians, nursing staff, management, technical staff, implementers) through regular ongoing meetings [64]. Provider (e.g., clinicians and nurses) participation in the planning and design of the system was a major contributor to successful implementation, partly because it facilitated ownership of the program during the planning stage [59].

##### Gain leadership and management support and commitment

In 31 % (*N* = 15/48) of studies, leadership or management support and commitment were identified as vital factors for success and sustainability [55, 58, 76]. Obtaining management support for a telemedicine program and formal commitment from leaders was important as part of the preparation for change, especially before allocating resources for implementation [62, 75]. The alignment between administrative and clinical leadership was also deemed important for success [82]. Engaging in frequent communication with leaders throughout the change process is reported to be critical to maintain implementation “buy-in” and also helped tailor implementation strategies [60].

##### Identify champions

Telemedicine champions are described as “enthusiastic individuals who initiate and promote the uptake of telehealth services” [22]. A little under a third (*N* = 15/48) of the studies in our review reported that telemedicine champions are essential to securing successful telemedicine participation and uptake. These telemedicine champions played a role in promoting (e.g., awareness and education through example) [37, 55] and legitimized telemedicine, as well as building relationships with various stakeholders [58, 59, 74]. Selecting key staff members who were willing to actively participate in telemedicine programs and fundamentally believe in the concept of telemedicine was found to influence other people as well as support buy-in [59]. However, a few studies reported a drop in telemedicine activity when these champions left the role or organization [37], suggesting that the reliance on a sole champion in uptake may be detrimental to the long-term development and sustainability of telemedicine services [45].

##### Engage partners and stakeholders

Clinical providers, staff and administrators in health organizations typically have high workloads and multiple competing priorities, which influenced their engagement in new projects [49]. Yet, engaging key stakeholders at the beginning of a telemedicine implementation project, and bringing them together to understand the current need for, and challenges of, implementing telemedicine services was a necessity [38, 44, 48, 49]. Half (*N* = 24/48) of the articles in our review identified the involvement or engagement of key partners and stakeholders during the establishment of telemedicine projects or services. Engaging stakeholders (i.e., nurses, clinicians) in the design process of the system also facilitated ownership of the service [48]. Frequent communication with sites through emails, regular phone calls, in-person visits and attending formal and informal community conferences was found to help gain stakeholder involvement in new services [49]. Engagement involved building relationships, collaborations and alliances with a range of stakeholders including local communities, outlying clinics, external partners (e.g., regional and national health authorities or government agencies) and telemedicine experts [44, 48, 59, 67, 74].

##### Develop and articulate a clear, simple vision

Six studies mentioned developing a specific vision for the telemedicine service. One study reported having a long-term vision for a pilot project [38], others mentioned having a clear and simple vision as a key factor for successful implementation of telemedicine [55, 66]. Having a vision shared by different partners and stakeholders also helped facilitate the change [79].

#### Phase 1. Preparing for change – operational practices

In addition to these strategic practices, four operational practices have been identified as important for optimal telemedicine implementation. The first two practices provide an understanding of how to assess the opportunity or problem motivating the change; while the next two practices relate to the operational side of selecting and supporting a guiding change coalition.

##### Conduct a needs assessment

Fifteen studies reported the importance of conducting a needs assessment before tackling a complex intervention such as telemedicine. Understanding the characteristics, needs and expectations of telemedicine was important for designing a telemedicine solution that is compatible with end users, providers and the organization [44, 49]. Paying attention to the context and needs of clinics and providers was important when tailoring the implementation strategy [60, 72]. This process involved engaging stakeholders [81], as well as evaluating organizational readiness and the readiness of each partnering site, which provided the groundwork for understanding the needs of the organization [37, 72].

##### Assess compatibility of telemedicine equipment and applications

Ten studies reported the importance of assessing the compatibility of telemedicine when implementing new services. This practice took into account the other services being provided, the existing infrastructure and technology and the appropriate location for the equipment, as well as assessing the compatibility of telemedicine solutions with local work practices and processes [42, 45, 55, 67, 75]. This practice was found to be key determinants of the acceptance and effective integration of telemedicine into usual work processes [42].

##### Assign coordinating roles

Poor coordination and scheduling between local and distant sites are frequent and serious issues that undermine the establishment of telemedicine services [57]. Having assigned telemedicine coordinators at both participating telemedicine sites to aid connections during clinics significantly contributed to the success and greater use of telemedicine initiatives [37, 71]. Twenty studies identified the importance of coordinators for facilitating telemedicine services. Coordinators played an important role in preparing the organization for change [43, 51], scheduling and integrating telemedicine activities into clinical workflow patterns [56], liaising with participating stakeholders [49], troubleshooting technical issues [18, 56] and overseeing quality control of the service [56]. Frequent communication with telemedicine coordinators was shown to be vital in sustaining a telemedicine program, with one study reporting that telemedicine services ceased when the coordinator left [37]. Successful long-standing telemedicine programs normally included formally defined role descriptions that clearly specify telemedicine work duties, indicating that telemedicine is a formal part of regular work routines [44].

##### Ensure adequate resources

Workplace readiness for a new telemedicine system requires having adequate resources to support the telemedicine services. Fifteen of 48 studies identified the need for additional resources when integrating telemedicine services into existing practices. Studies often reported that telemedicine consultations require more resources than conventional consultations [42, 55], including additional time for nurses to prepare and participate in teleconsultations. In most cases, it was necessary to complete a formal workflow analysis to assign the appropriate number of staff members to support a new telemedicine service. This included technical support [45, 61], help-desk support [80] and administrative support [55].

#### Phase 2. Managing change – strategic practices

Five strategic practices were identified for managing the change. These practices focused on communicating the vision about a telemedicine service, mobilizing energy for change, empowering others to act and monitoring and strengthening the change process.

##### Communicate changes and understanding of telemedicine

Almost 50 % of the reviewed articles identified communication and/or dissemination of information about the changes and the benefits and limitations of telemedicine which was required in establishing a new service. Communication included sharing successful examples of telemedicine programs by conducting demonstrations or presentations to key stakeholders [36, 59] to convey the benefits and limitations of it [40, 42]. Participants of telemedicine projects also reported that providing information such as background information, clinical protocols and contact information as to be very useful for planning and implementing telemedicine clinics [42]. Raising community or population awareness was another suggested communication strategy to address resistance and sustain telemedicine services [40, 45, 68]. Understanding and realizing the benefits and advantages of telemedicine was understood to contribute to the success of a telemedicine program [40, 42].

##### Gain stakeholder trust, acceptance and buy-in

Half of the telemedicine studies (*N* = 24/48) reported provider, patient/user and community trust, acceptance and buy-in as key elements in establishing services, which directly impacted on the success or failure of a telemedicine service [54, 57]. Trust was relevant in multiple referents including in the technology being used [42], the trusting relationships amongst those providing the service, as well as trust of users and patients in the service [55, 61]. Establishing face-to-face relationships with rural community providers through site visits and starting conversations and talking through concerns about telemedicine generated trust among stakeholders [61, 62, 70]. Similarly, meetings with management, clinical and evaluation staff to address concerns and issues about the change were also useful practices to gain acceptance, trust and buy-in [43, 60]. Site visits also enhanced specialists’ understanding of the local clinical context [70] by learning about the cultural needs, historical, legal and political issues unique to specific communities [42, 49]. As confidence and familiarity was gained with the system, providers and patients were more likely to accept telemedicine as another modality for clinical consultations [37, 41, 42].

##### (Continue to) Engage partners and stakeholders

A few studies (*N* = 7/48) noted that ongoing engagement of key stakeholders throughout the change process was important to re-affirm the true value of the project, to gain regular feedback and provided an opportunity to discuss the challenges involved [43, 49, 64, 81].

##### Facilitate ownership of the service

A small number of studies (*N* = 8/48) identified the importance of ensuring ownership of a telemedicine service. One study noted that providing hospitals with the freedom of when and how the telemedicine service would be utilized, facilitated local ownership of the program [43]. Empowering others by engaging them in a shared leadership role also facilitated the ownership of a service [66].

##### Monitor change and maintain flexibility

Continuous or periodic feedback through reporting systems or regular meetings was identified as important for monitoring the change and refining the service offering (*N* = 23/48). These practices enabled clinical teams involved in telemedicine to tailor the services to meet clinical needs and continuously improve the program [75, 81]. Paying attention to the context and needs of clinics and providers was important when tailoring the implementation strategy and facilitated the adjustment of resources [60]. Maintaining flexibility involved an iterative process between the stakeholders (e.g., facilitators, providers and coordinators) to tailor the service [60, 63]. These practices involved trial-and-error (e.g. what technologies to use and different clinic work pathways) and acceptance of mistakes when implementing a plan [63, 78].

#### Phase 2. Managing change – operational practices

Two operational practices have been identified as crucial for managing the change, which related to developing and promoting change-related knowledge and ability. Providing training and education, as well as developing work protocols and processes facilitated work related changes.

##### Provide training and education

Providing training and education was identified as central to successful telemedicine adoption in the majority (*N* = 33/48) of articles reviewed. Training was normally delivered during the implementation of telemedicine to ensure maximum utility [50, 56, 78]. Training included how to use and troubleshoot equipment [46, 49] and how to perform consultations through the technology [52, 80]. Ongoing training and regular site checks were required to maintain telemedicine programs, especially in facilities with high staff turnover [41, 53, 67]. When dealing with rural and remote communities, cultural factors and communications styles also informed the training initiatives for providers [49].

##### Develop new work processes, protocols and procedures

The majority of articles (*N* = 31/48) also identified the need to develop new workflow processes, guidelines, and clinical protocols when implementing telemedicine services [40, 42, 43, 47]. These studies highlighted that it was necessary to customize or change existing workflow to accommodate the use of telemedicine [36, 49, 68]. Clear communication of protocols and guidelines between sites [57] and having adequate telemedicine information to develop clear precise operating procedures [42, 44] were considered critical to success. Linkages between traditional and new work models of service delivery contributed to the integration of telemedicine [45].

#### Phase 3. Reinforcing change – strategic practices

Two strategic practices were identified in this review to help sustain long-term change. These included practices that related to institutionalizing the change.

##### (Continue to) Engage partners and stakeholders

This practice (*N* = 7/48) emphasized the need to continue engaging partners and stakeholder throughout the change process. Preparing interim reports about the telemedicine services (e.g., number of consultations) [38], having ongoing meetings to discuss the progress of the program [49, 81], as well as providing ongoing training and education [67] were ways to continue engaging stakeholders to reinforce the change.

##### Evaluate the changes and maintain flexibility

To ensure the sustainability of telemedicine programs, a number of studies (*N* = 14/48) reported the need to evaluate the service after the preliminary implementation stage. Obtaining ongoing feedback from users helped refine the usability of telemedicine and the maintenance of compatibility of the technology and applications with the organisational needs in the long term [77]. Evaluating the change was done through provider and patient/staff feedback and satisfaction ratings, reviewing work-flow output, evaluating patient outcomes, quantification of the efficiency and assessing the capacity of telemedicine operations, as well as conducting a cost analysis [38, 39, 49, 57].

Figure 1 shows our CM practice framework, which captures a consolidated overview of the CM practices identified in our review. It depicts a process approach in preparing, managing and reinforcing change, which shows the ongoing work and process to sustain a new telemedicine service through a suite of CM practices used by various health care practitioners.

## Discussion

This scoping review brings together for the first time a comprehensive picture of the different ways practitioners have applied CM practices when implementing telemedicine services. Table 3 presents a summary of CM practices and examples reported in the telemedicine literature in this review, as well as the identified articles that mention the application of CM practices.

Through our review, we draw out three key insights that contribute to the literature on telemedicine implementation. First, most practitioners only considered CM practices retrospectively after particular issues had emerged, indicating a *reactive orientation* to dealing with implementation issues. A reason behind this may be the lack of appreciation and understanding of the complexity of CM processes and the practices that support it, as well as limited knowledge about how to lead, plan and implement organizational change [34]. People normally underestimate the work involved in implementing change and it often falls to busy front-line health care practitioners to facilitate change [83]. Recognizing the amount of work and tasks required to undertake change and having a dedicated coordinator with the necessary CM skills and knowledge to facilitate and implement change is important to ensure full time attention and dedication to the change process [20, 83]. In line with the broader CM literature, we advocate for a more *proactive approach* to implementing change. Those responsible for the change need to understand “how” to implement change, not just “what” needs to be changed [34].

Second, most studies identified the application of operational practices, such as developing new work processes, protocols and guidelines (69%), as well as providing training and education (72%). Training aligns with one of the most commonly used and mentioned CM activities, as reported in several MOMENTUM in-depth telemedicine cases [17]. This highlights the extent to which work practices and routines are impacted by the introduction of telemedicine. Integrating telemedicine is often challenging and disruptive to existing practices [16]. These disruptions place new demands on the organization and the people involved in delivering the telemedicine services, who often report that telemedicine consultations require more resources (e.g., time, new roles) than conventional consultations [84]. However, our review identified that only a quarter of studies reported on ensuring sufficient resources for telemedicine implementations. Adequate resourcing and support are essential to both drive and sustain change [17, 85, 86], however such resourcing is often difficult to obtain in the context of health care organisations with constrained resources [83]. Resources to sustain a service normally include an initial investment for the early deployment of a service and resources for on-going operations [17]. To ensure sustainable change, trade-offs may have to be made, such as the redeployment or redirection of scarce resources toward the new work activities of telemedicine services [85].

Third, we note that several studies in our review focus on either strategic *or* operational practices, but rarely include both, indicating scope to apply a more holistic CM approach when implementing telemedicine. When insufficient focus is given to strategic factors, efforts will be wasted on operational issues with little alignment between management practices to reconfigure work processes and address the changes in day-to-day operations [34]. Similarly, when too much focus is given to operational factors at the expense of strategic considerations, it is unlikely the required assistance, commitment and acceptance from stakeholders, sites and partners, will be achieved [20]. Without a critical mass of support for the operational change [87], implementation is unlikely to be successful. A combination of both strategic and operational practices is required to guide and support people throughout the process of change and ensure the sustainability of implemented services.

### CM practices that are not commonly reported in telemedicine studies

Based on current CM literature and models [20, 88, 89], the following CM practices have not been commonly reported in telemedicine implementation studies: 1) anticipate, and identify gaps and areas of resistance; 2) integrate change management plan into a project plan and; 3) celebrate success and short-term wins.

Resistance to adopting telemedicine services is a common barrier found in implementation studies of telemedicine [8, 25, 90], yet there is very little reported about how to effectively deal with resistance. It is vital for a CM strategy to include activities that anticipate, work through and manage resistance across each of the three phases of the CM process [88, 91] (see Fig. 1). CM plans have to be tailored to the types of resistance encountered, which requires assessing who might resist the change and for what reasons [88, 91]. According to Kotter & Schlesinger the four most common reasons why people resist change are: 1) people believe that the change will result in them losing something of value; 2) lack of trust and awareness about the implications of the change; 3) having a different assessment of the change (e.g., a belief that the change will incur more costs than benefits for themselves and the organization) and; 4) low tolerance for change [88]. Some ways to overcome resistance have already been identified in this review, such as training and education, engaging stakeholders and management support to facilitate the change process. However, these are often only applied in the initial phases of the implementation. Few managers realize that these CM practices need to be used to address resistance throughout the course of the change process.

The change management literature identifies other ways of dealing with resistance, such as leveraging and engaging senior leaders *throughout all phases of the change process* [34], influencing their direct reports and thus acquiring organizational commitment and support. While gaining leadership support was identified in a little over a quarter of the articles on telemedicine implementation in our review, the role of leadership was largely restricted to the initial first phase of the implementation process. Yet leaders play a crucial role in persuading and directing people throughout the change process [17, 92, 93], which starts by setting the stage for acceptance, framing the preliminary plan, managing the mood of the organization and reinforcing new behaviors and routines [93]. The CM literature makes clear that leaders need to play a visible role in guiding and supporting the change throughout all phases of the process [86]. According to Prosci’s benchmarking reports since 1998, active and visible leadership or sponsorship is the strongest contributor to the success of change initiatives [94]. Similarly, the MOMENTUM report highlights the importance of leadership through a ‘champion’ as a critical success factor [17]. Successful champions are normally in a position of authority or influence in the organization and can mobilize resources to implement and sustain a telemedicine service [17].

Creating a CM plan and strategy is a key practice identified in telemedicine review studies [16]. However, integrating CM and project management is rarely considered in telemedicine implementation projects. Project management and CM have different methodologies that are complementary and mutually supportive of each other, with each contributing to a higher likelihood of successful implementation of projects [89, 95]. When project management and CM are integrated the efforts of both can be focused on a single objective, risks can be more proactively identified and mitigated, project activities (i.e., technical and people) can be aligned and lastly, the flow of information can be more effectively used and managed (e.g., feedback about usage and adoption) [89].

Celebrating success and recognizing short-term or small wins are recognized as success factors prescribed in common models of change and an important step for reinforcing change [19, 20], yet rarely considered when establishing telemedicine services based on our review. These CM practices help maintain morale and encourages progress toward longer term change objectives, builds support and provide positive and public acknowledgement to reinforce change [19, 96]. Recognizing short-term wins can also help convince those who are skeptical about the change that it is viable and may prompt others to buy-in [20, 96].

Our review has some limitations related to our search strategy. First, we focused on articles published within the past 10 years in recognition of the fast-moving nature of technology to support telemedicine. While this ensures our review reflects current practice, there may have been other CM practices reported in earlier studies that are not represented in this review. Second, given that the last database search was conducted in June 2019, articles published since then will not be included. Third, our search may not have identified all relevant published studies or all CM activities, particularly where authors did not report all practices used during the implementation of telemedicine. Fourth, there may be articles relevant to our review within the grey literature, which were excluded as our search focused only on peer-reviewed studies. Fifth, while we followed Arksey and O’Malley’s framework for scoping reviews by adopting a broad search during study identification, there are additional search terms that could have been incorporated, which may have yielded further relevant studies. Sixth, limiting our search to include English-only articles may have resulted to excluding some relevant studies.

Finally, given limited resources and having initially commenced this scoping review during the doctoral dissertation of the first author, only one reviewer was systematically involved in all aspects of the search process but this was complemented by team discussions throughout the screening phase to assess abstracts, which did not clearly meet the inclusion and exclusion criteria, as well as full-text eligibility. Nonetheless, dependence on one reviewer may have led to some studies being missed. Given the clarity of our research aims and a defined inclusion and exclusion criteria, the reviewer’s dependence on interpretation regarding eligibility of studies would have been lessened [97]. While single screening has been used for some systematic reviews [97] and other scoping reviews (e.g., [98, 99]), we recognize that a dual approach in the screening and study identification process would have strengthened the results [100]. We are confident, given the broad inclusion criteria and the methodology described, that we have provided a more comprehensive and broader overview of what and how CM practices have been used in telemedicine implementation than has existed previously and drawn out important insights and learnings.

## Conclusions

Given the high volume of published telemedicine case reports, we were expecting a higher number of studies to report on the CM practices and strategies used during implementation. Yet what we found was that, while many studies report on the challenges or barriers encountered when implementing change or the CM practices that would have been beneficial to use in hindsight, only 48 studies reported on the practices and strategies actually used to facilitate implementation and adoption of telemedicine services.

Based on our scoping review, we suggest a need for a process-based approach which comprehensively deploys a combination of strategic and operational practices when managing change efforts. Instead of focusing on barriers and facilitators of change, we encourage future telemedicine [8] research to examine the change processes and practices used to achieve successful implementation, particularly those practices that address the cultural and people issues, given many barriers to adoption center around attitudes and behaviors towards change [8, 23]. This scoping review is a starting point for approaching the implementation of telemedicine as a *process of change* and we hope it will encourage future telemedicine research to explore and recognize the importance of effective CM practices not only in telemedicine services, but also in the implementation of other health care service projects more broadly.

## Data Availability

Not applicable

## Abbreviations

CM: Change management
ICTs: Information communication technologies
